# Aquatic Biodiversity in the Amazon: Habitat Specialization and Geographic Isolation Promote Species Richness

**DOI:** 10.3390/ani1020205

**Published:** 2011-04-29

**Authors:** James S. Albert, Tiago P. Carvalho, Paulo Petry, Meghan A. Holder, Emmanuel L. Maxime, Jessica Espino, Isabel Corahua, Roberto Quispe, Blanca Rengifo, Hernan Ortega, Roberto E. Reis

**Affiliations:** 1 Department of Biology, University of Louisiana at Lafayette, Lafayette, LA 70504, USA; E-Mails: tiagobio2002@yahoo.com.br (T.P.C.); arbeitenschnell@yahoo.com (M.A.H.); e.maxime@hotmail.fr (E.L.M.); 2 The Nature Conservancy and Museum of Comparative Zoology, Harvard University, MA 02138, USA; E-Mail: ppetry@tnc.org; 3 Museo de Historia Natural, Universidad Nacional de San Marcos, Lima, Peru; E-Mails: jessicaadela@hotmail.com (J.E.); mycorahua@yahoo.es (I.C.); rquispe73@yahoo.com.mx (R.Q.); brengifo@fondoamericas.org.pe (B.R.); hortega.musm@gmail.com (H.O.); 4 Pontifícia Universidade Católica do Rio Grande do Sul, Porto Alegre, RS, Brazil; E-Mail: reis@pucrs.br

**Keywords:** freshwater fishes, geodispersal, species diversity, species richness, stream capture, tropical rainforest, vicariance

## Abstract

**Simple Summary:**

The immense rainforest ecosystems of tropical America represent some of the greatest concentrations of biodiversity on the planet. Prominent among these are evolutionary radiations of freshwater fishes, including electric eels, piranhas, stingrays, and a myriad of small-bodied and colorful tetras, cichlids, and armored catfishes. In all, the many thousands of these forms account for nearly 10% of all the vertebrate species on Earth. This article explores the complimentary roles that ecological and geographic filters play in limiting dispersal in aquatic species, and how these factors contribute to the accumulation of species richness over broad geographic and evolutionary time scales.

**Abstract:**

The Neotropical freshwater ichthyofauna has among the highest species richness and density of any vertebrate fauna on Earth, with more than 5,600 species compressed into less than 12% of the world's land surface area, and less than 0.002% of the world's total liquid water supply. How have so many species come to co-exist in such a small amount of total habitat space? Here we report results of an aquatic faunal survey of the Fitzcarrald region in southeastern Peru, an area of low-elevation upland (200–500 m above sea level) rainforest in the Western Amazon, that straddles the headwaters of four large Amazonian tributaries; the Juruá (Yurúa), Ucayali, Purús, and Madre de Dios rivers. All measures of fish species diversity in this region are high; there is high *alpha* diversity with many species coexisting in the same locality, high *beta* diversity with high turnover between habitats, and high *gamma* diversity with high turnover between adjacent tributary basins. Current data show little species endemism, and no known examples of sympatric sister species, within the Fitzcarrald region, suggesting a lack of localized or recent adaptive divergences. These results support the hypothesis that the fish species of the Fitzcarrald region are relatively ancient, predating the Late Miocene-Pliocene (*c.* 4 Ma) uplift that isolated its several headwater basins. The results also suggest that habitat specialization (phylogenetic niche conservatism) and geographic isolation (dispersal limitation) have contributed to the maintenance of high species richness in this region of the Amazon Basin.

## 1. Introduction

Tropical America encompasses one of the greatest concentrations of organic diversity on Earth. In many groups of plants and animals species richness reaches a global zenith in the humid Neotropics [1,2]. The diversity of fishes in this region is especially impressive, with more than 5,600 species representing a majority of the world's freshwater fishes, and perhaps 10% of all known vertebrate species [3-5]. What is even more remarkable—and as yet unexplained—is how so many distinct evolutionary lineages of fishes can coexist in less than 0.002% of the Earth's total liquid water supply.

*How did so much biodiversity accumulate in such a small amount of total habitat space?* Or to paraphrase Hutchinson [6]: *Why are there so many kinds of Amazonian fishes*? Hutchinson sought explanations for species richness at the community level in terms of ecological mechanisms, hypothesizing for example that species-rich ecosystems with longer food chains and more niches are more stable, with a greater likelihood of persisting through time. Over the past half century many ecologists have followed Hutchinson [7] in the belief that species coexistence is enhanced by adaptive specialization, such that each species occupies a unique ecological niche or functional role within the ecosystem [8,9]. Under this view, species richness arises from the action of natural selection to reduce competition [10,11].

An alternative view focuses on the role of geographic space in the formation of local and regional species pools [12,13]. The central idea is that richness (numbers of species) and taxonomic composition of assemblages at local and regional scales are hierarchically interrelated [14,15]. This is because the species that inhabit local assemblages are recruited from the regional pool, and the regional pool is itself the sum of all the local assemblages [16-19]. This geographic view is consistent with the macroevolutionary perspective that net rates of diversification within a geographic region are a balance of differential rates of speciation, extinction and dispersal [20-23]. Indeed, the fish species composition of a given locality in lowland Amazonia is generally influenced by all three of these processes [24].

Most ecological studies on the formation of local species assemblages have focused on sessile organisms, especially plants [13,17,25-29]. Relatively less is known about the contributions of these processes in the formation of animal assemblages. Studies on animals have focused heavily on larval recruitment in marine assemblages [30-36], and environmental gradients and patchiness in terrestrial assemblages [37-43]. Freshwater faunas have also been explored for insights into the processes of community assembly [4,44-49]. Unlike terrestrial or marine environments, most freshwater habitats are spatially discrete at the landscape level, and the geographic range of most freshwater species is tightly linked to the course of modern and ancient river ways and watersheds [24,50-53].

**Figure 1:**
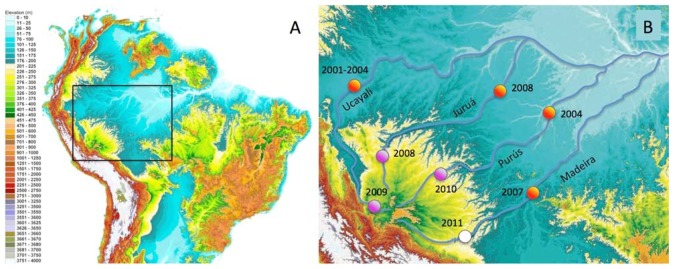
Topography of the Fitzcarrald Arch in southwestern Amazonia. (**A**) Elevational map of tropical South America from Shuttle Radar Topography Mission (SRTM) data in a Digital Elevation Model (DEM). Colors represent 25 m elevation intervals; blue-yellow transition at 200 m. (**B**) Close up of box in panel A. Purple circles indicate expedition locations (with year) between 212–310 m within the Fitzcarrald Arch. White circle indicates a forthcoming expedition. Orange circles indicate locations of comparable aquatic faunal inventories downstream conducted by the authors with colleagues.

In this paper we report the distributions of freshwater fish species restricted to discrete habitat types and river-courses in the Fitzcarrald Arch region in southwestern Amazonia. The Fitzcarrald region exhibits a unique geographic setting for studying the formation of species-rich aquatic Amazonian assemblages [54]. The region is a broad (∼400,000 km^2^), relatively low elevation (200–500 m elevation) topographic high, located entirely within the Amazon Basin to the east of the north central Andes (Figure 1). Importantly for the purposes of faunal comparisons, the tributary headwaters that drain the Fitzcarrald region are hydrologically isolated from one another, at least on ecological time scales, and the upstream portions of these basins (above 200 m) exhibit different habitat conditions from downstream portions in the Amazonian lowlands. The Fitzcarrald fish fauna therefore represents an excellent system in which to explore the roles of habitat specialization and geographic isolation in the formation of local species assemblages in diverse tropical aquatic ecosystems.

## 2. Results and Discussion

### 2.1. Comparisons between Basins and Habitats

**Figure 2:**
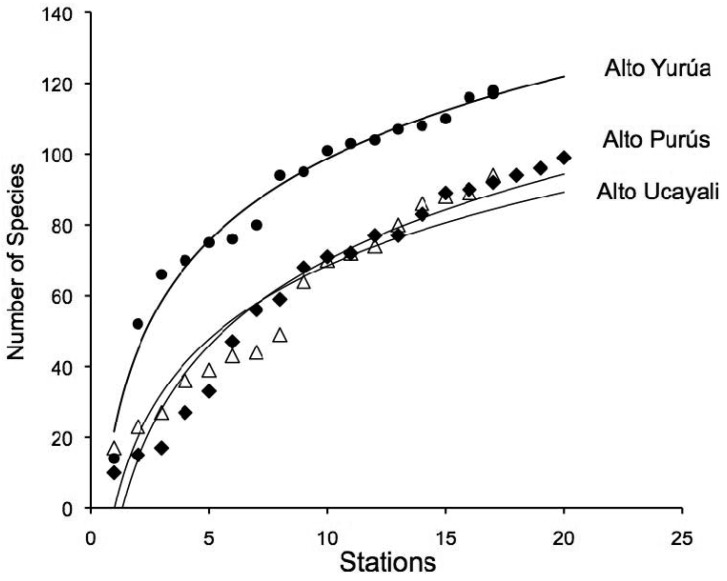
Accumulation curves of fish species collected in three expeditions to the interior drainages of the Fitzcarrald Arch, Peru. Each expedition involved about 20 field days during the period of low water (July) in the Alto Yurúa (2008), Alto Ucayali (2009) and Alto Purús (2010) basins. Note the accumulation curves do not approach asymptotic values.

A total of 208 morphospecies were positively identified from materials collected in the Fitzcarrald region (Figure 1, purple circles). Identifications were based on catalogued vouchers with associated color digital images and cross referenced tissue samples (Appendix 1). This represents a 76% increase over the 118 fish species previously documented with vouchers from this region [55-65]. The taxa listed in Appendix 1 represent approximately 4.5% (208 of 4,581) of the freshwater fish species of tropical South America, in an area spanning about 2.6% (400,000 of 15,400,000 km^2^) the total land surface area occupied by this fauna [5]. Species accumulation curves for each of the three expeditions are reported in Figure 2. These accumulation curves do not approach asymptotic values, and the species richness numbers reported in this study underestimate the actual values for these basins.

**Figure 3:**
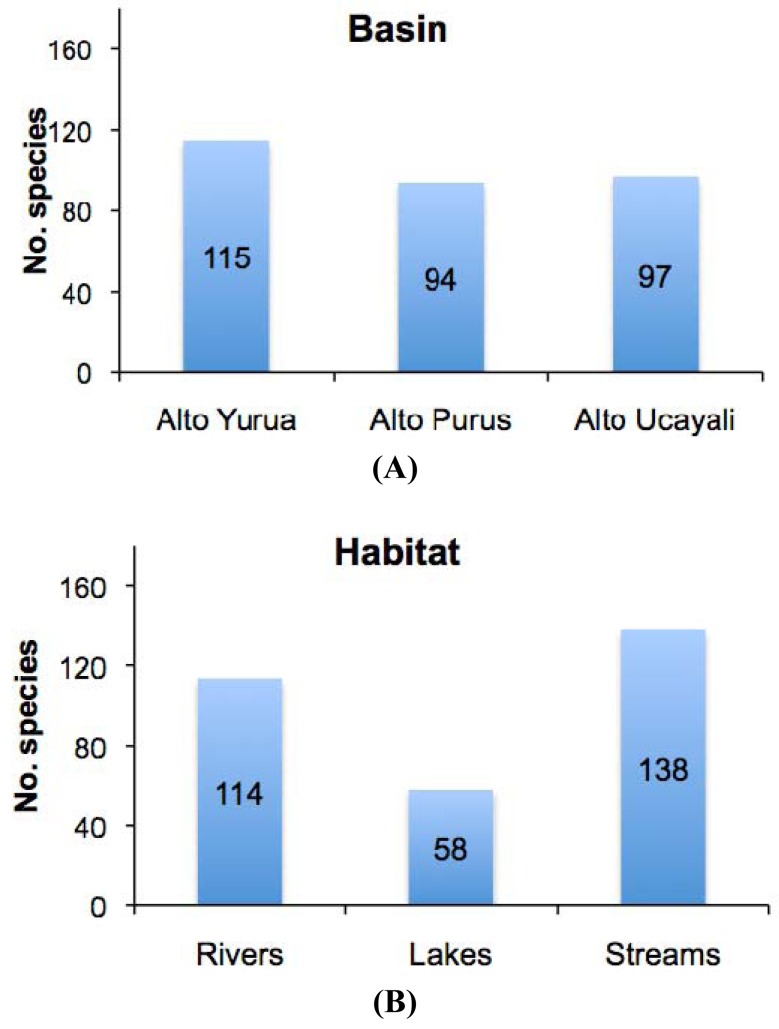
**(A)** Total species richness recorded in each basin. **(B)** Total species richness recorded in each habitat type.

**Figure 4:**
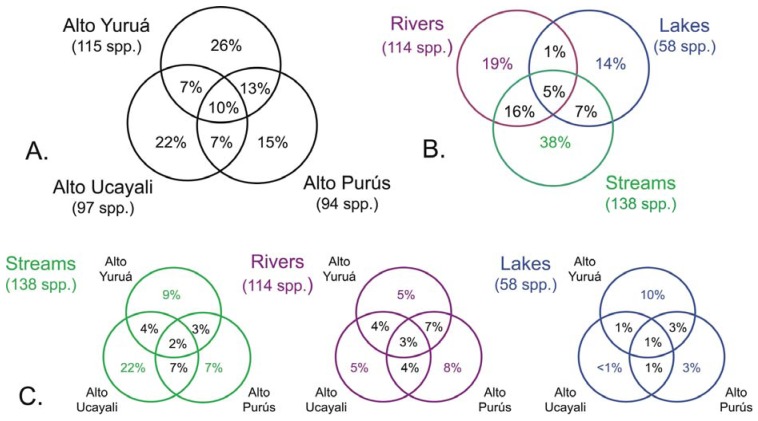
Venn diagrams summarizing shared fish species compositions of subregions (river basins) and major habitat types. (**A**) Among basins, habitats pooled. (**B**) Among habitats, basins pooled. (**C**) Among basins by habitat. Data presented as percentages of total Fitzcarrald species pool (208 species) to facilitate comparisons.

The results of this study showed high levels of geographic isolation among, and ecological specialization within, the tributary basins. The species richness values reported in each of the three basins were all very similar, each representing about half that of the regional species pool (Figure 3(a)); Alto Yuruá with 115 species (55%), Alto Ucayali with 97 species (46%), and Alto Purús with 94 species (45%). The taxonomic compositions (*i.e.*, species names) of the faunas were however very different; most species (133 species, 64%) collected were restricted to a single basin, 56 species (27%) were shared by two basins, and only 21 species (10%) were widespread in all three basins (Figure 4). These differences are reflected in the high *gamma* diversity between upland basins, which averaged 165 (79%) species turnover. The fish species encountered in upland localities within the Fitzcarrald Arch also differ substantially from localities of the same rivers further downstream (e.g., Ucayali, Juruá, Purús), where floodplain habitats dominate the regional diversity [56,57,63,66-68]. Local species richness (alpha diversity) of floodplain sites declines noticeably in all three of the basins draining the Fitzcarrald examined so far, from 70–100 species at downstream sites (100–120 m) to fewer than 30 species at upstream sites (>200 m).

**Figure 5:**
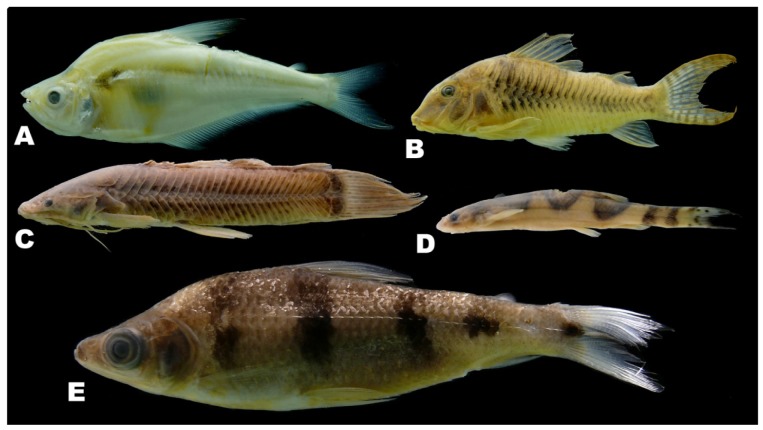
Examples of species collected only in the Alto Yuruá. **(A)** *Roeboides affinis* (70 mm). **(B)** *Corydoras stenocephalus* (54 mm). **(C)** *Callichthys callichthys* (59 mm). **(D)** *Pseudostegophilus nemurus* (64 mm). **(E)** *Schizodon fasciatus* (86 mm).

**Figure 6:**
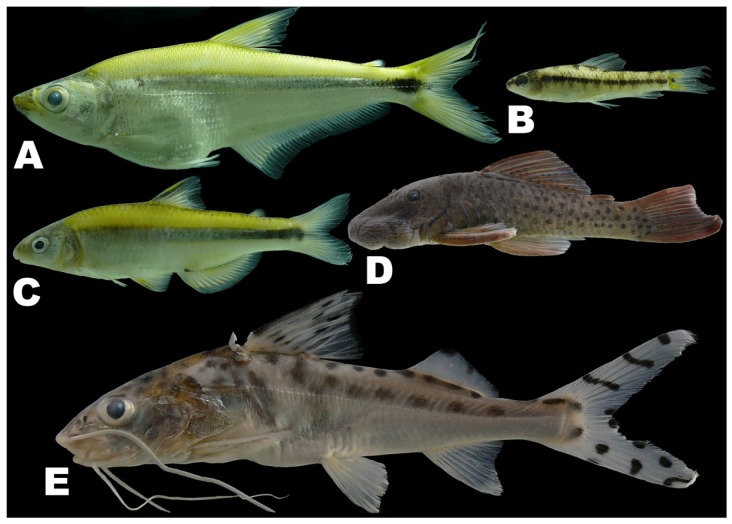
Examples of species collected only in the Alto Ucayali. **(A)** *Acestrocephalus boehlkei* (79 mm). **(B)** *Geryichthys sterbai* (26.7 mm). **(C)** *Attonitus ephimeros* (48 mm). **(D)** *Chaetostoma lineopunctatum* (52 mm). **(E)** *Pimelodus pictus* (78 mm).

**Figure 7:**
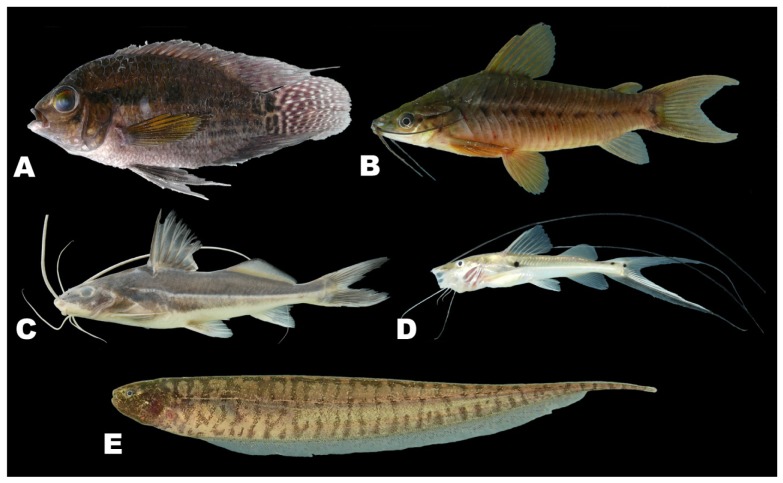
Examples of species collected only in the Alto Purús. **(A)** *Cichlasoma boliviense* (79 mm). **(B)** *Dianema longibarbis* (73 mm). **(C)** *Pimelodus sp.* 2 (112 mm). **(D)** *Platysilurus mucosus* (87 mm). **(E)** *Brachyhypopomus cf. beebei* (56 mm).

**Figure 8:**
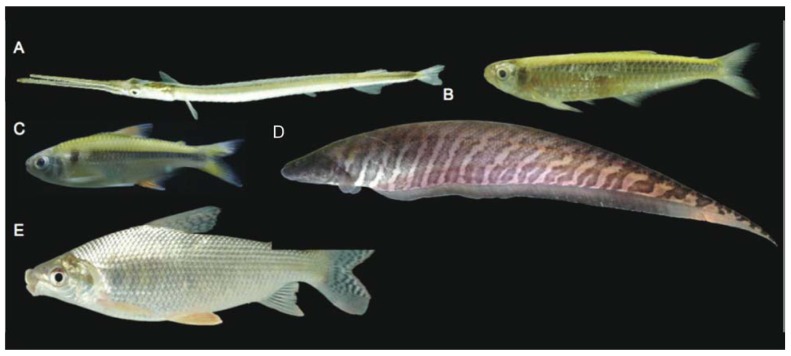
Examples of species collected in all three basins. **(A)** *Pseudotylosurus angusticeps* (73 mm). **(B)** *Clupeacharax anchoveoides* (67 mm). **(C)** *Knodus orteguasae* (46 mm). **(D)** *Gymnotus carapo* (280 mm). **(E)** *Prochilodus nigricans* (190 mm).

There was by contrast comparatively high heterogeneity in the species richness of the three habitats sampled, with a total of 114 species (55%) in rivers, 58 species (28%) in lakes, and 138 species (66%) encountered in streams. A large majority of the species (144 species, 69%) were stenotopic, that is restricted to a single habitat, and only 11 species (5%) were eurytopic, found in all three habitats. There were many more species in the lotic habitats (flowing waters of rivers and streams; 151 species, 72%) than in the non-riverine habitats (lakes and streams; 115 species, 46%) or floodplain habitats (rivers and lakes; 71 species, 34%). Examples of species collected only in the Alto Yuruá, Alto Ucayali, and Alto Purús basins are shown in Figure 5, Figure 6 and Figure 7. Five of the 21 species found in all three basins are depicted in Figure 8.

The high degree of habitat specialization in the Fitzcarrald is reflected in the high *beta* diversity (species turnover between habitats), which averaged 135 species (65%). A turnover of approximately half the species between adjacent habitats may seem remarkable, given the intimate proximity and physical contiguity of the waters draining small streams and oxbow lakes into the larger rivers, among sites separated by just a few tens to hundreds of meters. At least part of the reason for this high habitat specificity lies in the capacity of individuals in many floodplain species to move readily between sites over the course of the annual flood cycle, to find habitat conditions that better match their body size or other ecophysiological attributes [56]. Among the three basins, none of the habitats were found to share more than about 7% of the total species pool, and there were no substantial differences in actual numbers of species shared among the three habitats (Figure 4(C)). Another way to express this is that few species (5%) were found to be geographically widespread in any of the three habitats, and most were found restricted to a single basin (Figure 4(B)).

**Figure 9:**
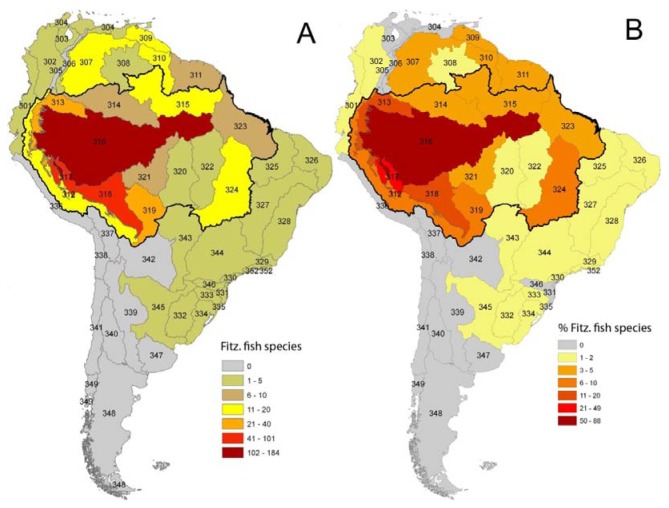
Geographic distributions of 208 Fitzcarrald fish species (FFS) among freshwater ecoregions (ER) of tropical South America. Black line indicates Amazon Basin watershed. **(A)** Total number of FFS per ecoregion. **(B)** Percent of FFS pool in that ecoregion. Data from Table 1. Note high numbers and proportions of FFS in the adjacent Amazonas Lowlands (ER 316; 88%), Ucayali-Urubamba (ER 317; 49%) and Mamoré-Madre de Dios (ER 318; 20%) Ecoregions.

**Figure 10:**
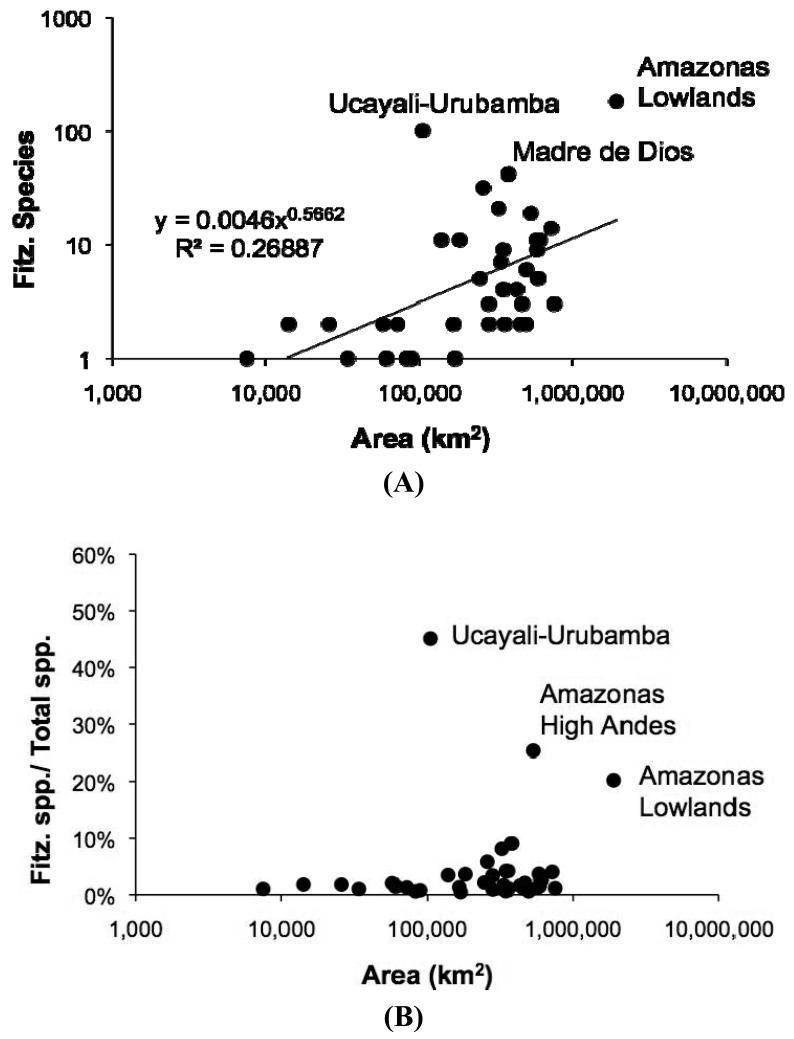
Species-area relationships for Fitzcarrald fish species (FFS) among the freshwater ecoregions of tropical South America. Species richness and area data from [5]. **(A)** Absolute numbers of FFS per ecoregion, showing high shared species composition with faunas in the adjacent Amazonas Lowlands, Ucayali-Urubamba, and Madre de Dios ecoregions. **(B)** Proportional representation of FSS per ecoregion, showing relatively high values in some nearby ecoregions, and low values in most other ecoregions.

As in any ichthyofauna, each fish species that inhabits the Fitzcarrald region exhibits a distinct geographic range, both within and beyond the Arch. The distributions of known Fitzcarrald fish species (FFS) among the freshwater ecoregions of tropical South America are presented in Table 1, and some generalities in these distributions are illustrated in Figure 9 and Figure 10. A large majority of the FFS (184 species or 88%) are present in the adjacent Amazonas Lowlands Ecoregion (ER 317) A large number of FFS are also shared with the adjacent Ucayali-Urubamba Piedmont Ecoregio (ER 312; 101 spp.; 49%), and Mamoré-Madre de Dios Piedmont Ecoregion (ER 318; 42 spp.; 20%) By contrast, relatively few FFS are present in other portions of tropical South America (e.g., Brazilia Shield).

The floodplain midline (thalweg) is a more accurate measure of fluvial separation among populations than are either Euclidean distance (“as the crow flies”), or river-channel distances. Euclidean distances as measured point-to-point on a map are inappropriate for assessing geographic separation among localities in the one-dimensional topological landscape of a drainage network [69]. Euclidean distances substantially underestimate the amount of habitat space required for dispersal by obligatory aquatic taxa, and the distance required for the transfer of material and energy through stream networks [70]. River channels, on the other hand, overestimate geographic separation among sites for riverine taxa in lowland Amazonian systems, as most if not all species that inhabit the deep channels also regularly use floodplain habitats on ecological time scales for forging and breeding [66,71].

Channel length (river distance) is a poor measure of fluvial spatial separation in lowland (below 200 m elevation) Amazonian ecosystems, where rivers exhibit high sinuosity (*i.e.*, have many meanders). Among the three rivers examined in this study, the total channel distances among sites range from 5,900 km (between Breu on the Alto Yuruá and Sepahua on the Alto Ucayali) to 6,765 km (between Sepahua and Pto. Esperanza on the Alto Purús), with the difference of 865 km representing about 13% that of the largest value (Table 2). Comparable ranges of distance estimates between these same sites are 21% for the floodplain midlines and 39% for Euclidean distances.

Channel length is generally well correlated with total basin area and floodplain area, and all three measures have been used as estimates of total habitat space in aquatic systems [72,73]. However, regional geomorphology can alter these relations, and channel length turns out to be a worse predictor of habitat space than does thalweg distance in highly meandering lowland Amazonian river systems. For example, the differences in channel length exhibited by rivers draining the Fitzcarrald Arch are relatively small as compared with other measures of basin size, such as total drainage area and water discharge [74,75]. This effect results from feedbacks of regional depositional hydrodynamics on channel length [76]. In the Yuruá, Purús and Madre de Dios basins the average channel sinuosity is proportional to floodplain width [77], which is very broad in the lower portions of the two shorter rivers, the Yuruá and Purús. These stretches are especially tortuous, attaining among the highest sinuosity values of any major rivers of the world, with values of about 2.3 [78,79].

### 2.2. Diversity Estimates and Sampling Biases

As of this writing we estimate the number of fish species in the interior of the Fitzcarrald region to exceed 300 species. This number is about one third more than the number used in the quantitative analyses of the present paper. The higher estimate was made by comparing species lists and photos presented in literature sources [55-65], and from examination of museum lots archived at the Natural History Museum of the University of San Marcos (MUSM), Lima. The quantitative comparisons of species diversity reported here were based on the more accurate and comparable species level identifications of our own collections, and not the higher species richness estimate based on literature references.

We emphasize the preliminary nature of the diversity estimates reported here, which are based on species presence and absence data only, and from limited sampling over a very small proportion of the Fitzcarrald region as a whole. The Fitzcarrald interior remains a remote wilderness landscape of lowland humid tropical forest with no roads, few air strips, and complex political interactions among the indigenous communities. Due to logistical constraints regarding access and transportation, we were unable to standardize the sampling protocol with sufficient rigor to collect species abundance data, or to test the effects sampling error on diversity estimates [80-85]. Quantitative data on fish species abundances in the Fitzcarrald region have to date focused on a limited number of commercially important, large-bodied, riverine species [59,86].

The Amazonian ichthyofauna in general remains incompletely documented, especially at the species level. A recent review calculated that, as of 2003, about 25% of Neotropical fish species known in museum collections were undescribed [87]. Much of the Amazonian lowlands is still a wilderness and the ichthyofaunas of many regions and river basins are either sparsely collected or, in some cases, almost entirely unknown [88]. There is also much poorer sampling of low order streams across the landscape, especially given the great proportion of the landscape they occupy, and the expectation for a relatively high species turnover among sites (gamma diversity) [89].

The sampling design of this study may also have introduced potential biases to the diversity estimates. In order to facilitate comparisons, collections were made during a standard period of low water in July when fishes are concentrated in lakes and channels, and not dispersed onto the floodplain. The lack of rain and mud also facilitates transportation by air, water and foot, and thereby increases the total number of localities, field days, stations, specimens and species that can be sampled within the limited time and other resources of a given expedition [56]. However, sampling at low water may under represent highly seasonal and migratory species (e.g., the pimelodid catfishes *Pseudoplatystoma* spp. and *Brachyplatystoma platynema*), as well as species associated with seasonally ephemeral habitats such as floating vegetation [71,90]. Interviews with local fishermen revealed the presence of several common lowland riverine species in upland portions of the Fitzcarrald rivers during the rainy season (Sept.-Dec.), that were not collected in our survey: e.g., potamotrygonid stingrays, the characiforms *Curimata aspera, Colossoma macropomum, Piaractus brachypomus, Raphiodon vulpinus*, and the perciform *Plagioscion auratus* [62,64]. Sampling at low water may affect gamma diversity estimates by under representing the number of species shared between floodplain habitats (rivers and lakes) due to seasonal lateral migrations. The anomalously low-water conditions encountered in the Alto Purús in 2010 as a result of a regional drought [91] may also have reduced the number of collection sites, fish abundances, and species diversity.

The small proportions of fish species shared among collections in the three basins of this study (Figure 4(C)) may reflect sampling error, dispersal limitation, or both. The very low level of species endemism recorded within the Fitzcarrald region as a whole suggests that dispersal has been important to the formation of the basin-wide fish faunas. Further, some species are expected to be absent from our collections do to perennially low abundances, or population variability in space or time (seasonal or annual variation). Other recorded absences may reflect real differences in absolute abundances. Some fish taxa (e.g., *Rivulus, Corydoras*) are widespread, abundant, and diverse in Amazonian lowlands, and really do seem to be absent or rare in the Fitzcarrald uplands. Among Gymnotiformes, the electric signals of *Gymnotus coropinae* and *Hypopygus lepturus* were never detected using a portable amplifier. As these species are usually common and abundant in lowland terra firme (non-floodplain) habitats, they also seem to be either absent or rare in the Fitzcarrald uplands. The absence of the callichthyids *Callichthys callichthys* and *Hoplosternum littorale* from many sites was also informative, as both species are usually common floodplain species in the lowland reaches of all the rivers draining the Fitzcarrald region, and neither had been previously reported from the upper reaches of these rivers [92,93]. Based on these experiences collecting in the Fitzcarrald uplands it is now possible to interpret the negative results as indicating real species absence, or at least rarity.

Some of the taxonomic patterns reported here may also be affected by the sampling gear, in particular our heavy reliance on nets (albeit of several kinds). Under sampled taxa may include certain pimelodid catfishes and apteronotid electric fishes inhabiting deep river channels, and species that take refuge in structurally complex habitats and substrates; e.g., doradid and auchenipterid catfishes in logs and logjams of streams and rivers; sternopygid and hypopomid electric fishes in rooted vegetation of floodplain lakes. Plant-based ichthyocides are widely used for subsistence fishing by indigenous peoples of the region, and were adventitiously employed in this study. However industrially produced rotenone is banned for scientific collecting in Peru and was not applied in this study. Although electrofishing is often useful in sampling structurally complex substrates, the apparatus is heavy and cumbersome to transport and use in remote settings that are often accessible by foot alone, and was not applied in this study.

### 2.3. Comparisons with Other Faunas

Despite the potentially important sampling biases described above, the fish faunas of the Fitzcarrald region appear to be composed of species recruited primarily from the exceptionally diverse Amazonian lowlands, and to a lesser extent from the fish faunas of the Ucayali and Madre de Dios basins (Table 1). There are high numbers (and proportions) of Fitzcarrald species present in the adjacent Amazonas Lowlands (184 species or 88%), Ucayali-Urubamba (101 species or 49%) and Mamoré-Madre de Dios (42 species or 20%) ecoregions (Figure 9). The absolute numbers of Fitzcarrald fish species is highest in the adjacent Amazonas Lowlands, Ucayali-Urubamba, and Madre de Dios ecoregions (Figure 10(A)), and proportional representation of Fitzcarrald fish species is highest in the Ucayali-Urubamba, Amazonas High Andes, and Amazonas Lowlands ecoregions (Figure 10(B)).

**Figure 11:**
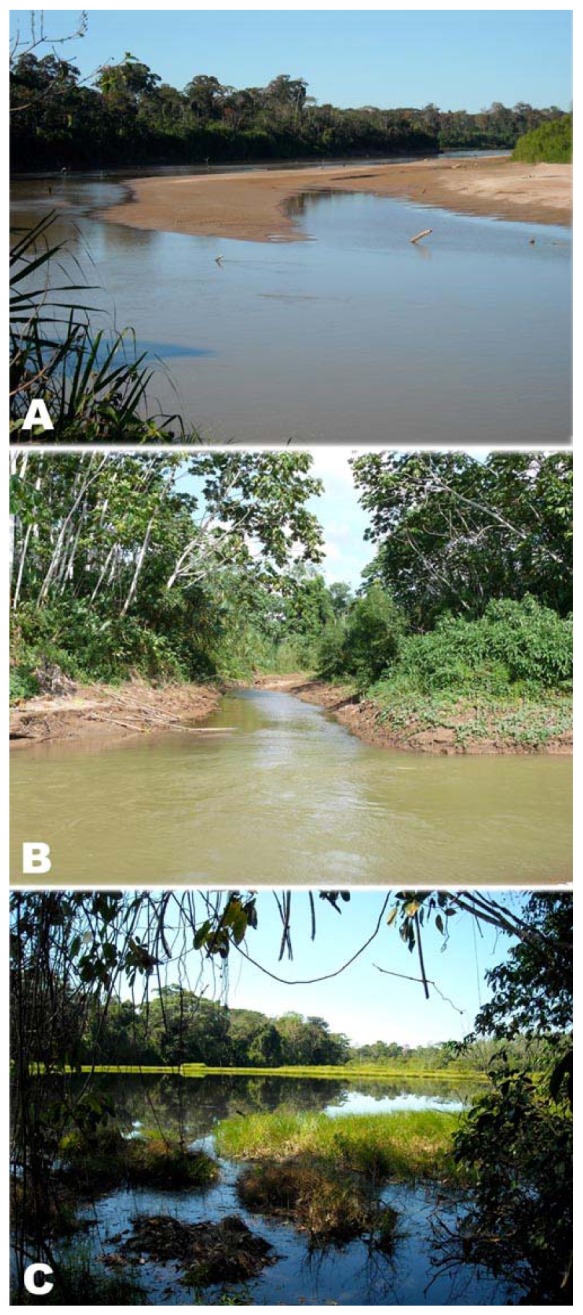
Examples of the three major aquatic habitats sampled in the Fitzcarrald region. **(A)** Channel and flooded beaches of the Rio Purús at San Marcos (9°53′S 70°52′W). **(B)** A stream emptying into the Mishaua river in the Urubamba basin (11°13′S 72°58′W). **(C)** A floodplain oxbow lake (Cocha Supiri) in the Purús basin (9°58′S 70°55′W).

Upstream localities of the Fitzcarrald fish fauna appear to be relatively depauperate in comparison with downstream localities of comparable habitat in the same river basin [56,57,63,67,68]. Some common and widespread Amazonian fish taxa that are to date entirely absent from the Fitzcarrald uplands include: *Lepidosiren, Pellona, Semaprochilodus, Metynnis, Myleus, Brycon, Acestrorhynchus, Hydrolicus, Hypopygus, Microsternarchus, Rhamphichthys, Hoplosternum, Cichla* and *Colomesus*. The relative rarity or perhaps complete absent of these species in the Fitzcarrald uplands may be due in part to local ecological conditions. The margins of streams and rivers traversing the interior of the Fitzcarrald region are dominated by sandy or muddy beaches at low water, with little rooted and almost no floating aquatic vegetation (Figure 11). Further, the upland river channels are relatively shallow, and the floodplains substantially narrower, with fewer and smaller oxbow lakes than in lowlands. In combination these conditions are not favorable to many fish groups specialized to inhabit lowland Amazonian floodplains and deep river channels (e.g., the *várzea* guild of Serrasalminae; see Figure 3 in [94]). The pattern of fish species richness falling off with elevation has been reported in other regions of tropical South America sampling over similar elevational gradients [95-98].

### 2.4. Endemism

Very few fish species are known to be endemic within the Fitzcarrald region. At present we know of only three species; the gymnotid electric fish *Gymnotus chaviro* [99], the characin *Phenacogaster capitulatus* [100] and the cichlid *Bujurquina eurhinus* [101]. However, the task of documenting species endemism requires much more spatial information than does that of documenting species richness, as endemism requires positive knowledge of where species are both present and absent, and extensive geographic sampling with fine resolution at the alpha taxonomic level [5,102]. The actual patterns of species endemism in the remote and relatively poorly explored rivers of Western Amazonia may not be known for many years.

Given the relatively poor understanding of the FFS fauna, there is reason to suspect that both taxonomic and geographic factors contribute to the conclusion of little or no endemism within the Fitzcarrald region. The observed lack of endemism may in fact be real, but limited (or apply most strongly) to the floodplain portion of the fauna that was most intensively sampled, or perhaps to the peripheral portion of the Fitzcarrald region that was sampled at elevations below 310 m. Low levels of species endemism are indeed widely observed in other floodplain areas of lowland Amazonia [5]. Under this view it remains possible that some fish species are restricted to non-floodplain (terra firme) habitats of the Fitzcarrald, especially in the interior uplands (310–500 m), and that the elements of the fauna intercepted by the current study represent primarily the most common and widespread forms.

Nevertheless, the low endemism of fish species in the Fitzcarrald region does appear to be a genuine feature of the fauna, and we anticipate this result will be robust in the face of future discoveries [5]. We suggest, cautiously, that the data currently in hand really do indicate low levels of fish species endemism within the Fitzcarrald. The taxonomic and geographic understanding of this region is really not so much worse than many other areas of comparable size the Western Amazon, some of which really do exhibit distinct a species composition (ERs 313, 317, 318). In addition, the sampling intensity of this study, focused on the periphery of the Fitzcarrald (212–310 m), is likely to have intercepted at least some species endemic to the interior. The sampling station (Quebrada El Dorado on the Mishaua river) closest to the point of highest elevation (536 m) within the Fitzcarrald region lies at a distance of about 110 km as the crow flies. This is a relatively small distance in the context of Amazonian fish species whose ranges commonly extend over many hundreds of km in linear dimensions. Unsampled endemic species would therefore have to have very restricted geographic distributions, low abundances, or both. Lastly, analysis of geospatial information from DEM and high resolution satellite (Google Earth) images indicate an absence of pronounced physical barriers to dispersal (e.g., a fall line), or discrete climatic differences, between the peripheral(lower) and interior (upper) portions of the Fitzcarrald region. Indeed elevations associated with changes in fish species compositions usually occur above 500 m in other upland regions of tropical South America [5].

### 2.5. Paleogeographic Considerations

In addition to the unique ecological conditions alluded to in the Introduction, the Fitzcarrald Arch represents a unique geological situation, containing the only watersheds within the Amazon Basin for which reliable estimates are currently available regarding the timing of headwater basin separation [5,24]. The uplift of the Fitzcarrald Arch is dated by geophysical and paleontological data to the Late Miocene-Pliocene (9–3 Ma) [103-106]. Such information on the timing of river basin separation provides minimum age estimates for calibrating genetic divergences among populations or species inhabiting each of the newly isolated basins [52,107-114].

Analyses of radiometric and biostratigraphic data indicate that the Fitzcarrald region changed from a depositional to an erosional setting during the period of the Late Miocene to Pliocene epochs (*c.* 9–3 Ma.) [103,115-118]. Sedimentological analyses show the switch occurred in association with the transition from mid-Miocene (Quechua phase) faulting to Pliocene (Diaguita phase) compressional deformation [103]. Radiometric Argon-Argon dating of two volcanic tuffs from the Solimões Formation were dated to *c.* 9 and *c.* 3 Ma. [116]. Mammalian biostratigraphy confirms these age estimates, as the top of the Solimões Formation (Chapadmalan Stage) has no mammal fossils of North American origin [119]. These data suggest that sedimentation in the Solimões Formation ceased before the rise of the Isthmus of Panama and the onset of the Great American Biotic Interchange *c.* 3.5 Ma. Lastly, molecular dating of divergences among aquatic mammal and fish populations [120,121] suggest Late Miocene-Pliocene dates for the separation of the Upper Madeira from other Amazonian basins.

Although rising to only modest elevations, the Fitzcarrald uplift contributed to the Miocene fragmentation of the north flowing Subandean Foreland Basin, and to the reorganization of the fluvial net that forms the modern east-flowing Amazon Basin [104-106,122-124]. The low elevation and unconsolidated sediments of the region has resulted in a relatively continuous history of stream capture events across its several watershed divides, as the principle direction of stream flow shifted from generally NW to NE [125].

The most likely candidates for divergence in allopatry across low-lying Amazonian watersheds are clades (monophyletic groups) of species with spatially restricted and non-overlapping geographic ranges, and clades of species possessing small body size, stenotopic (ecologically narrow) habitat preferences and limited dispersal capacities. A recent meta-analysis of diversification in Amazonian fishes examined patterns in 33 taxa (genera or tribes) with sufficiently dense taxon and geographic sampling to test hypotheses of allopatric divergence [24]. Among these taxa no species-pair has yet been identified that matches criteria for divergence in allopatry across one or more of the Fitzcarrald watersheds.

Taxa that stand as candidates for possible species-level divergence across one or more of the Fitzcarrald watersheds include the characins *Characidium* (8 species), *Knodus* (7 species), *Moenkhausia* (5 species), and *Odontostilbe* (4 species), and the armored catfishes *Ancistrus* (7 species), *Hypostomus* (6 species), and *Panaque* (4 species). There is to date no phylogenetic information on the species of these taxa from the Fitzcarrald region. Available phylogenetic information on several other taxa is however inconsistent with such an hypothesis; e.g., *Leporinus* with four species [126], and *Gymnotus* with two species [99]. Genetic and phenotypic data on the armored catfish *Chaetostoma lineopunctatum* show incipient within-species divergence between the Ucayali and Madre de Dios basins [127], but this species really has an Andean distribution, and this example may not therefore represent divergence across a Fitzcarrald watershed. In general, the alpha taxonomy and systematics of all the taxa mentioned above remain very poorly understood. Phylogenetic and phylogeographic studies of several Fitzcarrald fish taxa are currently underway using molecular data to examine species-level divergence across Fitzcarrald watersheds.

## 3. Experimental Section

All collections were made between 212–310 m above sea level in portions of three major Amazonian basins draining the Fitzcarrald region (Table 3). The collections were made during the period of low water (July) when fish biomass is more concentrated and field sites are more accessible. The overall sampling strategy was to examine the relative roles of geography (river basin) and habitat in constraining the species composition of local assemblages. A total of 53 upstream localities were sampled within the Fitzcarrald region in 48 field days over three project years (Figure 1(B); Table 3).

Collections were made in three major types of environments: channels and flooded beaches of large rivers (>40 m wide on straight runs at low water), small rivers and streams, and floodplain oxbow lakes (Figure 11). All collecting stations were georeferenced (latitude, longitude, altitude) using GPS, and habitats were documented with high resolution digital photographs and written descriptions. Abiotic attributes such as water temperature, pH and electrical conductivity were measured using a HI 98129 Multimeter (Hanna Instruments). Collections were made using standard ichthyological gear, including seine nets (5 and 10 m, 5 mm between knots), dip nets, cast nets, and hook and line. Electric fishes were located with the aid of a portable amplifier [128].

All specimens collected were identified to morphospecies, and exemplars of each morphospecies set aside as vouchers for a standardized reference collection. Exemplar specimens were measured (standard length in mm), digitally photographed, and individually labeled with a field number attached to GPS coordinates and water quality data. Fish sizes are reported in millimeters standard length, or length to end of anal fin for Gymnotiformes. Tissue samples were excised using a sterilized scalpel and preserved in 100% ethanol in 1.8 mL vials with o-ring sealed caps, and then stored in a cool location at the base camp before transport to the laboratory. All voucher specimens were fixed in 10% formalin for at least 48 hours in a closed Nalgene container or covered flat plastic tray (for larger specimens), and later transferred to 70% ethanol. Fish specimens were collected under permits issued annually for each expedition from the Peruvian Ministry of the Environment, and all specimens were catalogued at the Museum of Natural History, University of San Marcos (MUSM), Lima. Images and collection data of all species are available from the project website at: www.ucs.louisiana.edu/~jxa4003/Alto%20Purús.html.

Quantitative comparisons of taxonomic composition (*i.e.*, species lists) were made among each of the three headwater basins, and between habitat types within a basin, using standard diversity measures [129-131]. *Alpha* diversity was calculated as simply the number of species recorded at a local site. Changes in species composition were assessed using *beta* and *gamma* measures of diversity. *Beta* diversity between habitats was calculated as: β = (*S*_1_ − *c*) + (*S*_2_ − *c*), where *S*_1_ and *S*_2_ are the species richness values of adjacent habitats, and *c* is the number of species in common between two habitats. *Gamma* diversity between river basins was calculated as: γ = *S*_1_ + *S*_2_ − *c*. Qualitative comparisons of the taxonomic composition between upstream (>200 m) and downstream (<200 m) sites within a basin made using species presence and absence data from other faunal inventory projects conducted by the authors and colleagues (Figure 1(B); orange circles). Interregional comparisons were made by recording each species of the Fitzcarrald fish fauna as either present or absent in each of the freshwater ecoregions of tropical South America using a published dataset and methods [5]. Species presence data was based on confirmed identifications from catalogued museum records, photographs in literature reports, or in consultation with numerous specialists (see Acknowledgements). Ecoregion boundaries were defined primarily by hydrographic (river basin) limits, with some boundaries also defined using other landscape or physiographic discontinuities [132].

Distance between sampling locations were estimated using the Path function in Google Earth [133]. Distances were measured as Euclidean length, river channel length, and floodplain midline length. Floodplain midline distances are equivalent to the thalweg (valley line) of fluvial geomorphology signifying the deepest continuous line along the middle of a watercourse [134]. Sinuosity (meander ratio) is a measure of how much a river channel deviates from the shortest thalweg path. Sinuosity is calculated as river distance (=channel length) divided by floodplain midline distance, where the sinuosity of a straight river is 1.0 and higher ratios indicate greater sinuosity [72,135]. Sinuosity is highly correlated with stream velocity and sediment load, both quantities of which are maximized at a sinuosity of 1.0. Rivers with more meanders run more slowly and drop more sediment on the floodplain.

## 4. Conclusions

Accumulating evidence suggests that Neotropical fish species diversity is ancient, with regional species pools accumulating over tens of millions of years and over a geographical arena spanning multiple hydrogeographic basins. Similar patterns are also emerging for many elements of the Neotropical terrestrial biota, including amphibians [136-140], reptiles [141-144], birds [114,145-149], and mammals [119,150,151]. In other words, the exceptional species richness of local Amazonian assemblages is generally not the result of local diversification. Rather, species accumulated at a continental scale and over geological time frames. These patterns of biodiversity and biogeography at the species level have been observed in most if not all Neotropical fishes [5], and other taxa in which biotic diversification is bound by ecology and physiology to landscape history [152]. In almost all taxa, sympatric species assemblages are of polyphyletic origin, and comprise species with distributions that span far outside the area of sympatry and predate the Pleistocene climate oscillations; *i.e.*, they are not the result of recent *in situ* radiations [24,52].

Comparisons of taxa distributed across the headwater tributaries of the Fitzcarrald region provide biogeographic tests for the generality of models on the formation of regional species pools. Interspecific phylogenies and phylogeographic (intraspecific) data do not in isolation provide rigorous tests for alternative hypotheses concerning the geography of speciation, because of the lability of geographical ranges and the lack of correlation between the role of adaptive processes and geographical mode of speciation [153-155]. However, concordances in species-area relationships and phylogeographic patterns among multiple taxa do help illuminate the sequence and relative timing of hydrological events (e.g., separation and merging headwaters) that may strongly influence the diversification of aquatic taxa [52,156].

The quantitative results of this study are sensitive to the finite sampling effort permitted by logistical constraints of working in such a remote region. Although the results reported in this study must be tested by additional sampling, the available data suggest that the ichthyofaunas of the Fitzcarrald region are not the result of localized or recent adaptive radiations.

## Figures and Tables

**Table 1:** Distribution of known Fitzcarrald fish species (FFS) among the freshwater ecoregions of tropical South America. Data for 208 FFS in 39 ecoregions extending over about 13.9 million km^2^. Ecoregions not listed have no FFS. Minimum number of total fish species per ecoregion from Albert *et al.* (2011, table 2.1). Note the greatest number of FFS in Amazonas Lowlands (184 species, or 88% of FFS pool). Note also FFS constitute a higher proportion of the total fish fauna in the Ucayali-Urubamba ecoregion (45%) than any other ecoregion.

**Ecoregion**	**Area km^2^**	**FFS**	**% FFS**	**Total Species**	**FSS/Total species**
301–Atrato & NW Pac. Coast	282,596	2	1%	215	1%
302–Magdalena & Sinu	357,251	2	1%	182	1%
303–Maracaibo	88,785	1	0%	127	1%
304–Caribbean Coast-Trinidad	169,425	1	0%	216	0%
306–Orinoco Piedmont	82,491	1	0%	168	1%
307–Orinoco-Llanos	575,142	11	5%	809	1%
308–Orinoco-Guiana Shield	348,090	4	2%	637	1%
309–Orinoco Delta & Coastal	138,602	11	5%	315	3%
310–Essequibo	182,512	11	5%	301	4%
311–Eastern Guiana	336,492	7	3%	413	2%
312–Amazonas High Andes	530,073	19	9%	75	25%
313–Marañon-Napo-Caqueta	258,909	32	15%	548	6%
314–Rio Negro	496,301	6	3%	668	1%
315–Amazonas Guiana Shield	605,130	11	5%	430	3%
316–Amazonas Lowlands	1,909,012	184	88%	910	20%
317–Ucayali-Urubamba	104,605	101	49%	224	45%
318–Mamoré-Madre de Dios	378,174	42	20%	463	9%
319–Guaporé-Itenez	326,437	21	10%	258	8%
320–Tapajós-Juruena	429,427	4	2%	244	2%
321–Madeira Brazilian Shield	349,019	9	4%	214	4%
322–Xingu	463,772	3	1%	142	2%
323–Amazonas Estuary	580,379	9	4%	243	4%
324–Tocantins-Araguaia	717,332	14	7%	346	4%
325–Maranho Piauí	354,584	4	2%	95	4%
326–Mid-Northeast. Caatinga	281,757	3	1%	88	3%
327-São Francisco	592,794	5	2%	181	3%
328–Mata Atlantica	454,322	2	1%	180	1%
329–Paraiba do Sul	57,726	2	1%	97	2%
330–Ribeira do Iguape	25,731	2	1%	110	2%
331–South Brazilian Coastal	33,979	1	0%	97	1%
332–Lower Uruguay	246,932	5	2%	230	2%
333–Upper Uruguay	71,820	2	1%	153	1%
334–Laguna dos Patos Basin	165,638	2	1%	150	1%
335–Tramandaí-Mampituba	7,506	1	0%	97	1%
343–Paraguay	492,705	2	1%	332	1%
344–Upper Parana	751,513	3	1%	258	1%
345–Subtropical Potamic Axis	586,319	5	2%	331	2%
346–Iguaçu	60,664	1	0%	68	1%
352–Fluminense	14,053	2	1%	110	2%

**TOTAL**	13,907,999	208	100%	4,581	5%
**MIN**	7,506	1	0%	68	0%
**MAX**	1,909,012	184	88%	910	45%
**AVG**	356,615	14	7%	275	5%

**Table 2:** Distance estimates (km) between downstream collection sites in three basins of the Fitzcarrald Arch, southeastern Peru. AU = Alto Ucayali at Sepahua; AP = Alto Purús at Pto. Esperanza; AY = Alto Yuruá at Breu. See the text for definitions and methods.

**Basin pair**	**Euclidean**	**Thalweg**	**Channel**	**Mean Sinuosity**
AU-AY	180	3,767	5,900	1.57
AU-AP	295	4,548	6,765	1.49
AU-AM	442	5,348	6,305	1.18
AY-AP	223	3,614	6,665	1.84
AY-AM	505	4,412	6,205	1.41

**Avg.**	329	4,338	6,368	1.50
**Stdev**	140	693	352	0.24
**min**	180	3,614	5,900	1.18
**max**	505	5,348	6,765	1.84
**max-min**	325	1,734	865	0.67
**range %**	0.64	0.32	0.13	0.36

**Table 3:** Summary of locality data of collections to date in the Fitzcarrald region of Southeastern Peru under NSF-DEB 0741450. Vouchered lots = reference collection of morphospecies with digital images, tissue samples, and associated water quality data. Total cataloged lots at MUSM, Lima.

**Basin**	**Base (Year)**	**Lat. Lon.**	**Alt. range**	**# stations**	**Field days**	**Vouch. Lots**	**Tot. Cat. Lots**
Yuruá	Breu (2008)	09°31′S 72°45′W	232–260 m	17	20	272	880
Ucayali	Sepahua (2009)	11°08′S 73°02′W	273–310 m	20	16	369	1,255
Purús	Esperanza (2010)	09°46′S 70°43′W	212–259 m	17	12	175	975

**Total**				**53**	**48**	**816**	**3,110**

## Appendix

**Appendix 1:** Distribution of fish species by tributary basin and habitat type in the Fitzcarrald region of Southeastern Peru. Note the similar species richness values among the three tributaries (94–115 species; avg. 102 ± 12.3 species), and the greater heterogeneity of species richness values among the three habitats (58–138 species; avg. 100 ± 39.1 species).

Family	Genus species	Alto Yurúa	Alto Ucayali	Alto Purús	# basins	River	Stream	Lake	# habitats
Belonid	*Pseudotylosurus angusticeps*	X	X	X	3	X	X		2
Anostomid	*Leporellus vittatus*	X			1	X			1
	*Leporinus friderici*	X	X	X	3	X		X	2
	*Leporinus striatus*	X			1	X			1
	*Leporinus trifasciatus*		X		1	X			1
	*Leporinus yophorus*	X		X	2	X			1
	*Schizodon fasciatus*	X			1			X	1
Characid	*Acestrocephalus boehlkei*		X		1	X	X		2
	*Aphyocharax pusillus*	X		X	2	X	X	X	3
	*Astyanacinus multidens*		X		1	X	X		2
	*Astyanax abramis*	X	X		2	X	X		2
	*Astyanax bimaculatus*		X	X	2	X	X	X	3
	*Astyanax maximus*		X		1	X	X		2
	*Astyanax sp. 1*		X		1		X		1
	*Astyanax sp. 2*			X	1	X	X		2
	*Attonitus ephimeros*		X		1		X		1
	*Bryconacidnus sp*			X	1		X		1
	*Bryconamericus pachacuti*		X		1		X		1
	*Bryconamericus sp. 1*		X		1		X		1
	*Bryconamericus sp. 2*		X		1		X		1
	*Ceratobranchia obtusirostris*		X		1		X		1
	*Ceratobranchia sp.*			X	1		X		1
	*Charax sp.*	X	X		2		X	X	2
	*Charax tectifer*	X			1		X		1
	*Clupeacharax anchoveoides*	X	X	X	3	X	X		2
	*Creagrutus barrigai*	X		X	2	X	X		2
	*Creagrutus changae*		X		1	X	X		2
	*Creagrutus occidaneus*			X	1	X	X		2
	*Creagrutus pila*		X		1	X	X		2
	*Creagrutus sp.*		X		1		X		1
	*Ctenobrycon hauxwellianus*	X	X	X	3		X	X	2
	*Engraulisoma taeniatum*			X	1	X			1
	*Galeocharax gulo*	X		X	2	X	X		2
	*Gephyrocharax sp.*	X	X	X	3	X	X		2
	*Gymnocorymbus thayeri*	X			1			X	1
	*Hemibrycon jelskii*		X		1		X		1
	*Hemibrycon sp.*			X	1		X		1
	*Hemigrammus marginatus*		X		1	X	X		2
	*Knodus hypopterus*		X	X	2	X	X		2
	*Knodus orteguasae*	X	X	X	3	X	X		2
	*Knodus smithi*	X	X	X	3	X	X	X	3
	*Knodus sp. 1*	X	X		2	X	X		2
	*Knodus sp. 2*	X			1		X		1
	*Knodus sp. 3*	X			1		X		1
	*Knodus sp. 4*		X		1		X		1
	*Leptagoniates steindachneri*	X	X		2	X	X		2
	*Microgenys sp.*		X		1		X		1
	*Moenkhausia comma*	X			1		X		1
	*Moenkhausia dichroura*	X		X	2	X	X		2
	*Moenkhausia intermedia*		X		1	X	X	X	3
	*Moenkhausia oligolepis*	X	X	X	3	X	X		2
	*Moenkhausia sp.*			X	1	X	X		2
	*Mylossoma aureum*			X	1	X			1
	*Mylossoma duriventre*	X			1	X			1
	*Odontostilbe euspilura*		X		1		X		1
	*Odontostilbe fugitiva*	X	X	X	3	X	X	X	3
	*Odontostilbe sp. “P”*	X	X		2	X	X	X	3
	*Odontostilbe sp. 1*	X			1				0
	*Odontostilbe sp. 2*		X		1		X	X	2
	*Paragoniates alburnus*		X	X	2	X	X		2
	*Phenacogaster capitulatus*	X	X		2	X	X		2
	*Prionobrama filigera*	X		X	2	X	X		2
	*Prodontocharax melanotus*		X	X	2	X	X	X	3
	*Roeboides affinis*	X			1		X		1
	*Roeboides myersii*	X			1		X		1
	*Scopaeocharax* cf. *rhinodus*		X		1		X		1
	*Salminus sp.*			X	1	X			1
	*Serrasalmus rhombeus*	X	X		2		X	X	2
	*Serrapinnus sp. 1*	X		X	2		X	X	2
	*Tetragonopterus argentus*	X		X	2	X		X	2
	*Triportheus albus*	X		X	2	X	X		2
	*Triportheus angulatus*	X	X	X	3	X	X		2
	*Xenurobrycon heterodon*		X	X	2	X	X	X	3
Crenuchid	*Characidium fasciatum*	X			1	X	X		2
	*Characidium* cf. *purpuratum*		X		1		X		1
	*Characidium* cf. *steindachneri*		X		1		X		1
	*Characidium* aff. *zebra*		X		1	X	X		2
	*Characidium sp. 1*		X		1	X			1
	*Characidium sp. 2*		X		1		X		1
	*Characidium sp. 3*			X	1	X			1
	*Characidium sp. 4*			X	1			X	1
	*Geryichthys sterbai*		X		1		X		1
Curimatid	*Curimatella meyeri*	X			1		X		1
	*Cyphocharax* cf. *festivus*	X			1			X	1
	*Cyphocharax spiluropsis*	X			1			X	1
	*Potamorhina altamazonica*	X		X	2		X	X	2
	*Steindachnerina* cf. *dobula*	X		X	2	X	X		2
	*Steindachnerina guentheri*	X	X	X	3		X	X	2
	*Steindachnerina hypostoma*	X	X		2	X	X		2
	*Steindachnerina* aff. *insculpta*	X			1	X			1
	*Steindachnerina leuscisca*			X	1	X	X		2
Erythrinae	*Erythrinus erythrinus*	X			1			X	1
	*Hoplerythrinus uniaeniatus*	X			1			X	1
	*Hoplias malabaricus*	X	X	X	3		X	X	2
	*Carnegiella myersi*	X			1		X		1
Gastropelecid	*Thoracocharax stellatus*	X		X	2	X	X		2
Lebiasinid	*Copeina guttata*	X			1		X		1
Parodontid	*Paradon pongoensis*	X	X		2	X	X		2
Prochilodontid	*Prochilodus nigricans*	X	X	X	3	X	X		2
Engraulid	*Lycengraulis batesii*	X			1	X			1
	*Anchoviella carrikeri*		X		1	X	X		2
	*Rivulus sp.*	X	X		2		X	X	2
Apteronotid	*Apteronotus albifrons*		X		1		X		1
	*Sternarchorhynchus stewarti*	X			1	X			1
	*Sternarchorhynchus sp.*		X	X	2	X	X		2
Gymnotid	*Electrophorus electricus*	X		X	2		X	X	2
	*Gymnotus carapo*	X	X	X	3		X	X	2
	*Gymnotus chaviro*	X		X	2			X	1
	*Gymnotus ucamara*		X		1	X	X		2
Hypopomid	*Brachyhypopomus* cf. *beebei*			X	1			X	1
Sternopygid	*Eigenmannia virescens*		X		1	X	X		2
	*Sternopygus macrurus*	X		X	2	X	X	X	3
Cichlid	*Aequidens tetramerus*			X	1			X	1
	*Bujurquina* cf. *eurhinus*			X	1		X		1
	*Bujurquina robusta*	X	X		2	X		X	2
	*Cichlasoma amazonarum*	X			1			X	1
	*Cichlasoma boliviense*			X	1			X	1
	*Cichlasoma sp. 1*	X			1			X	1
	*Crenicichla proteus*		X		1		X	X	2
	*Crenicichla sedentaria*	X	X		2	X	X	X	3
	*Crenicichla semicincta*	X		X	2		X	X	2
	*Pachyurus schomburgkii*	X			1	X			1
	*Pachyurus cf. stewarti*			X	1	X	X		2
	*Plagioscion squamosissimus*			X	1	X			1
Achirid	*Apionichthys finis*	X	X		2	X			1
	*Hypoclinemus mentalis*	X			1	X			1
Aspredinid	*Bunocephalus coracoideus*		X		1		X		1
	*Pseudobunocephalus bifidus*	X			1			X	1
Auchenipterid	*Centromochlus perugiae*		X		1		X		1
Callichthyid	*Callichthys callichthys*	X			1			X	1
	*Corydoras aeneus*	X			1		X		1
	*Corydoras stenocephalus*	X			1		X		1
	*Lepthoplosternum altamazonicum*	X			1			X	1
	*Lepthoplosternum* cf. *stellatum*	X			1			X	1
	*Dianema longibarbis*			X	1			X	1
Cetopsid	*Cetopsis coecutiens*			X	1	X			1
Doradid	*Nemadoras sp.*	X			1	X	X		2
	*Trachydoras steindachneri*	X			1	X			1
Heptapterid	*Cetopsorhamdia phantasia*		X		1		X		1
	*Cetopsorhamdia sp.*			X	1	X			1
	*Chasmocranus sp.*		X		1		X		1
	*Imparfinis stictonotus*	X	X	X	3	X	X	X	3
	*Phenacorhamdia sp.*			X	1	X			1
	*Pimelodella* cf. *gracilis*	X			1	X			1
	*Pimelodella sp. 1*	X		X	2		X		1
	*Pimelodella sp. 2*	X		X	2	X	X		2
	*Rhamdia quelen*	X			1	X	X		2
Loricariid	*Ancistrus sp. 1*	X			1		X		1
	*Ancistrus sp. 2*	X			1		X		1
	*Ancistrus sp. 3*	X			1			X	1
	*Ancistrus sp. 4*		X	X	2		X		1
	*Ancistrus sp. 5*		X	X	2		X		1
	*Ancistrus sp. 6*		X		1		X		1
	*Ancistrus sp. 7*		X		1	X	X		2
	*Chaetostoma lineopunctatum*		X		1		X		1
	*Crossoloricaria rhami*	X			1			X	1
	*Farlowella kneri*		X		1		X		1
	*Farlowella nattereri*		X	X	2	X	X		2
	*Farlowella smithi*		X		1		X		1
	*Furcodontichthys* cf. *novaesi*	X			1			X	1
	*Hemiodontichthys acipenserinus*		X	X	2		X	X	2
	*Hypoptopoma thoracatum*	X			1			X	1
	*Hypostomus* cf. *emarginatus*	X	X	X	3	X	X		2
	*Hypostomus* cf. *pyrineusi*	X			1		X		1
	*Hypostomus pyrineusi*		X	X	2	X	X		2
	*Hypostomus unicolor*	X	X	X	3	X	X		2
	*Hypostomus sp. 1* (black dots)		X	X	2	X	X		2
	*Hypostomus sp. 2*		X	X	2		X		1
	*Lamontichthys filamentosus*	X		X	2	X	X		2
	*Lasiancistrus schomburgkii*	X	X		2	X	X		2
	*Limatulichthys griseus*	X		X	2	X	X		2
	*Loricaria sp.*	X	X	X	3	X	X		2
	*Loricariichthys sp.*	X			1		X	X	2
	*Panaque albomaculatus*		X		1	X			1
	*Panaque changae*	X	X	X	3	X			1
	*Panaque purusiensis*			X	1	X			1
	*Panaque schaeferi*			X	1	X			1
	*Peckoltia brevis*			X	1	X			1
	*Pterygoplichthys lituratus*	X			1			X	1
	*Pterygoplichthys punctatus*	X			1			X	1
	*Pterygoplichthys pardalis*			X	1			X	1
	*Rineloricaria lanceolata*		X		1		X		1
	*Spatuloricaria puganensis*	X		X	2	X			1
	*Sturisoma nigrirostrum*		X		1	X	X		2
	*Sturisoma sp. 1*			X	1	X	X		2
Pimelodid	*Brachyplatystoma juruense*	X		X	2	X			1
	*Brachyplatystoma rousseauxii*			X	1	X			1
	*Calophysus macropterus*	X	X	X	3	X			1
	*Cheirocerus eques*	X		X	2	X	X		2
	*Megalonema amaxanthum*	X		X	2	X			1
	*Megalonema platycephalum*		X	X	2	X			1
	*Pimelodus blochi*		X	X	2	X	X	X	3
	*Pimelodus ornatus*			X	1		X		1
	*Pimelodus pictus*		X		1	X			1
	*Pimelodus sp. 1*	X	X	X	3	X	X	X	3
	*Pimelodus sp. 2*			X	1	X			1
	*Pinirampus pirinampu*	X		X	2	X			1
	*Platystomatichthys sturio*	X			1	X			1
	*Platysilurus mucosus*			X	1		X		1
	*Sorubim lima*	X			1	X	X		2
Pseudopimelodid	*Batrochoglanis raninus*		X		1		X		1
	*Pseudopimelodus pulcher*	X		X	2	X	X		2
Trichomycterid	*Acanthopoma annectens*	X	X		2	X	X		2
	*Henonemus punctatus*	X			1	X			1
	*Plectrochilus sp.*	X			1	X			1
	*Pseudostegophilus nemurus*	X			1	X	X		2
	*Tridentopsis pearsoni*			X	1			X	1
Synbranchid	*Synbranchus madeirae*	X			1			X	1
	**Total**	115	97	94		114	138	58	
	**%Total**	0.55	0.47	0.45		0.55	0.66	0.28	
